# Mental fatigue decreases complexity: Evidence from multiscale entropy analysis of instantaneous frequency variation in alpha rhythm

**DOI:** 10.3389/fnhum.2022.906735

**Published:** 2022-10-26

**Authors:** Yawen Zhai, Yan Li, Shengyi Zhou, Chenxu Zhang, Erping Luo, Chi Tang, Kangning Xie

**Affiliations:** ^1^School of Biomedical Engineering, Air Force Medical University, Xi'an, China; ^2^Shaanxi Provincial Key Laboratory of Bioelectromagnetic Detection and Intelligent Perception, Xi'an, China; ^3^School of Electronics and Information, Xi'an Polytechnic University, Xi'an, China; ^4^Air Force Hospital of Western Theater Command, Chengdu, China

**Keywords:** mental fatigue, nonlinear analysis, complexity, multiscale entropy, auditory steady-state response

## Abstract

Mental fatigue (MF) jeopardizes performance and safety through a variety of cognitive impairments and according to the complexity loss theory, should represent “complexity loss” in electroencephalogram (EEG). However, the studies are few and inconsistent concerning the relationship between MF and loss of complexity, probably because of the susceptibility of brain waves to noise. In this study, MF was induced in thirteen male college students by a simulated flight task. Before and at the end of the task, spontaneous EEG and auditory steady-state response (ASSR) were recorded and instantaneous frequency variation (IFV) in alpha rhythm was extracted and analyzed by multiscale entropy (MSE) analysis. The results show that there were significant differences in IFV in alpha rhythm either from spontaneous EEG or from ASSR for all subjects. Therefore, the proposed method can be effective in revealing the complexity loss caused by MF in spontaneous EEG and ASSR, which may serve as a promising analyzing method to mark mild mental impairments.

## 1. Introduction

Mental fatigue (MF) represents a psychobiological state caused by prolonged periods of demanding cognitive activity (Tran et al., 2020). MF jeopardizes performance and safety through a variety of cognitive impairments, including decreased vigilance (Dimitrakopoulos et al., 2018). There is no standard definition of fatigue; clinically, fatigue is often defined as difficulty in performing voluntary activities (Chaudhuri and Behan, 2004). MF can sometimes be called cognitive fatigue (Persson et al., 2007).

Since MF impairs a variety of cognitive functions, it should present “complexity loss” in physiological signals, e.g., electroencephalogram (EEG), heart rate variation, according to complexity loss theory, which was initially proposed to define aging as a progressive loss of complexity in the dynamics of all physiologic systems (Lipsitz and Goldberger, 1992). Since it was proposed, complexity loss theory has been applied to various diseases, e.g., epilepsy (Lehnertz and Elger, 1995), heart failure (Costa et al., 2003), Alzheimer's disease (Dauwels et al., 2011), diabetes (Churruca et al., 2008), patients with vegetative state (Goldberger et al., 2002; Sarà and Pistoia, 2010), as well as in sleep studies (Shi et al., 2017; Li et al., 2021).

However, the studies are few and inconsistent concerning the relationship between MF and loss of complexity. For example, MF caused by watching 3DTV reveals significant complexity loss in occipital lobe in some scales (Chen et al., 2018). Another study shows increased complexity of EEG from subjects after performing days of intellectual competition (Zhang et al., 2021). Complexity is often measured by multiscale entropy (MSE) (Costa et al., 2002) and its variants (e.g., Wu et al., 2013; Shi et al., 2017; Han et al., 2020) in a variety of research fields by calculating sample entropy at multiple scales of the coarse-grained versions of the original signal.

Alpha frequency is important in cognition. On long-time scale, alpha frequency changes with age and certain diseases.

From early childhood up to puberty alpha frequency increases, but then starts to decline with age (Niedermeyer, 2005; Stomrud et al., 2010; Grandy et al., 2013). For adults, Köpruner et al. (1984) have found a linear relationship between age and alpha frequency. Some studies have reported diseases related decrease in alpha frequency, e.g., Alzheimer's disease (Klimesch et al., 1990; Babiloni et al., 2008), thalamocortical dysrhythmia (Llinás et al., 1999), chronic pain (Furman et al., 2020), or other neurological diseases (e.g., Torres et al., 1983; Moretti et al., 2007). In short-term scale, alpha power (or amplitude), varying far more salient with a task than alpha frequency does, is often used to identify mental fatigue (Wu et al., 2022), or to explain cognitive mechanisms (Haegens et al., 2022). Alpha frequency fluctuates with a cognitive demanding task, indicating that alpha frequency plays a role in the task (Klimesch et al., 1993; Angelakis et al., 2004).

In the previous study, we have found that alpha frequency fluctuates constantly in a nonlinear fashion by analyzing the complexity of instantaneous frequency variation (IFV) of alpha rhythms (Li et al., 2021). And it turns out that this complexity of IFV well-differentiates underlying cognitive states showing low complexity in sleep EEG than in wakeful EEG, which is in accordance with the complexity loss theory (Li et al., 2021).

In this study, We have analyzed the complexity of alpha rhythm and its IFV from spontaneous EEG, and from ASSR, which has been induced by stimulating the subjects with Don chirp sounds (Elberling et al., 2007) to test the hypotheses that MF may cause the complexity loss in EEG.

## 2. Materials and methods

### 2.1. Participants

Thirteen male college students, aged 20–22 years old (means = 21, standard deviation = 1.2), were recruited for this experiment. All the subjects met the following conditions, which were assessed by a questionnaire: no dementia; no significant changes on brain imaging; right-handedness; no history or evidence of psychiatric or neurological disease, cardiovascular disease, diabetes, thyroid disease, vitamin B12 deficiency, or substance abuse; normal vision or corrected normal vision; and being native Chinese. They were asked to ensure 8 h of sleep per day the week prior to the experiment, to abstain from food that might affect the excitability of the nervous system.

Informed consent forms were signed by all the subjects in this study. This study has been approved by the Institutional Committee of the Air Force Medical University.

### 2.2. ASSR stimulation

A chirp stimulus attempts to compensate for the cochlear traveling wave delay by aligning the arrival time of each frequency component in the stimulus to its place of maximum excitation along the basilar membrane (Elberling et al., 2007). The observed latency-frequency functions of the electrophysiological data can all be described by the same power function as suggested by (Anderson et al., 1971):


(1) $$\begin{array}{l} \tau =kf^{-d} \end{array}$$

where τ is the latency in seconds, *f* is the frequency in hertz (Hz), and *k* and *d* are constants. According to Elberling et al. (2007)'s study, *k* = 0.0920, *d* = 0.4356. ASSR can be elicited effectively with frequency range 40–50 and 80–100 Hz, and clinical applications often use the 40 or 80 Hz (Dobie and Wilson, 1998; Maki et al., 2009). We chose *f* = 80 Hz, which is far away from alpha and beta waves, to avoid possible interference of the stimulating frequency with the spontaneous EEG activity as well as mains interference around 100 Hz, which is the harmonic of 50 Hz. Broad band Don chirp signal was constructed by adding together a harmonic series of cosines with the frequencies, 160, 240, 320, …, 7,920 Hz. Huawei CM33 in-ear wired headphones were used to play the Don chirp sound.

### 2.3. EEG acquisition

EEGs of the subjects were collected from 16-scalp electrodes according to the international 10-20 system through a wireless device (model: NSW316, Neuracle, Changzhou, China). The raw EEGs were filtered with a band-pass filter from 0.01 to 100 Hz and sampled at 1,000 Hz with reference of CPz. Electrode impedance was kept below 5 kΩ throughout the recording.

### 2.4. Experimental protocol

**Figure 1:**
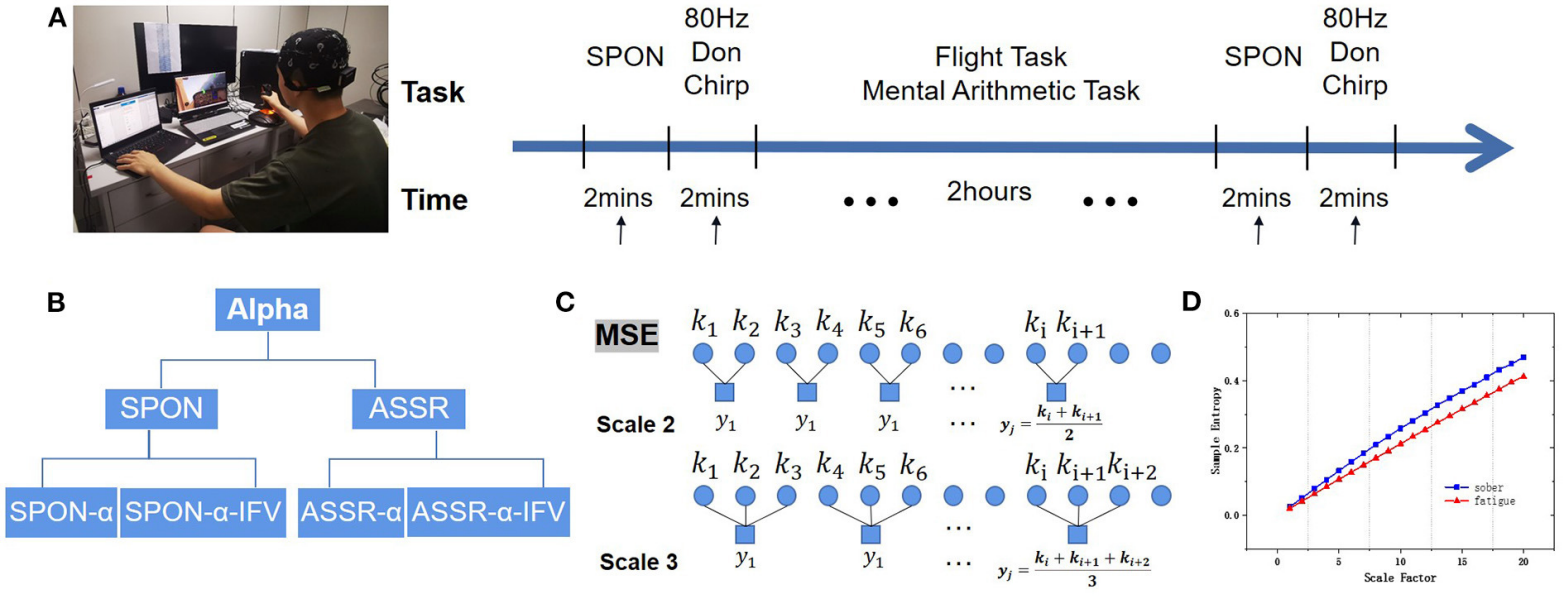
Experimental protocol. **(A)** Experimental flow diagram of EEG acquisition of spontaneous EEG (SPON) and auditory steady-state response (ASSR) in Sober and MF states. **(B)** Alpha rhythm extraction from SPON and ASSR with and without instantaneous frequency variation method (IFV), producing preprocessed four types of time series, SPON-α, SPON-α-IFV, ASSR-α, and ASSR-α-IFV, before they were analyzed by multiscale entropy methods (MSE) to compare Sober and MF states. **(C)** Schematic diagram of the coarse graining process, taking scale 2 and scale 3 as examples. **(D)** An example of MSE comparison.

Subjects were asked to repeat the airfield traffic pattern under visual flight rules in a simulated flight task inside an electromagnetically shielded room. The subjects were seated in a comfortable chair that supported their backs and heads in front of a computer monitor. Before flight, 2-min of spontaneous activity and 2-min of evoked activities by Don chirp sound were recorded with eyes closed. During the 2-h flight, the subjects were also asked to mentally solve 60 problems of additions or subtractions between two 3-digit numbers. After the flight task, 2 min of spontaneous activity and 2 min of evoked activities by Don chirp sound were recorded again with eyes closed. The Experimental Protocol is demonstrated in Figure 1.

### 2.5. Alpha rhythm extraction and IFV computation

Alpha rhythm was extracted from the spontaneous EEGs (SPON) or ASSR by a zero-phased fourth-order Butterworth band-pass filter with cutting-off frequencies 8 and 13 Hz. Alpha rhythm time series extracted from SPON and ASSR were named SPON-α and ASSR-α. Instantaneous frequencies were computed from alpha rhythm by Hilbert transform and then detrended to obtain the IFV (Li et al., 2021), resulting in time series SPON-α-IFV and ASSR-α-IFV, with data length 10,000 points.

### 2.6. Complexity measurement: Multiscale entropy analysis

MSE has been proposed to quantify the complexity of biomedical time series (Costa et al., 2002). In this paper, MSE is used to analyze the complexity of spontaneous EEG activities and ASSR in alpha band.

Refer to Costa et al. (2002) for details of MSE method. A short summary is as follows.

Assume that there is a discrete time series X of length N, *X* = [*x*_1_, *x*_2_, *x*_3_, ⋯ , *x*_*N*_]. The non-overlapping coarse graining process is defined as:


(2) $$\begin{array}{l} y_{j}^{\left(\tau \right)}=\frac{1}{\tau }\overset{j_{\tau }}{\underset{i={\left(j-1\right)}_{\tau +1}}{\sum }}x_{i},\;1\leq j\leq N/\tau \end{array}$$

where τ denotes the time scale, *j*_τ_ is the maximal scale, and *N*/τ denotes the length of each time series after coarse granulation. When τ = 1, the result of coarse-grained data is the original time series. In this study, *j*_τ_ was set to 20 following MSE convention (Costa et al., 2002). Figure 1C shows the coarse-grain process.

Next, the sample entropy is calculated for each time resolution corresponding to the MSE,


(3) $$\begin{array}{l} MSE\left(\tau \right)=SampEn\left(\tau ,m,r,n\right) \end{array}$$

where *m* represents the reconstruction dimension, *r* is the similarity tolerance, and *n* is the length of the data. *r* is calculated as *r* = *c*·σ, in which, σ is the standard deviation of the original sequence *X*_*i*_ and *c* is the tolerance factor, a percentage of σ with typical values between 0.1 and 0.25. In this study, *m* = 2, *c* = 0.15.

We define the complexity index (*C*) as the averaged MSE values over all scale factors (from 1 to 20):


(4) $$\begin{array}{l} C=\overset{20}{\underset{\tau =1}{\sum }}MSE\left(\tau \right)/20 \end{array}$$

We also define the complexity loss rate (*L*) as:


(5) $$\begin{array}{l} L=\frac{C_{before}-C_{after}}{C_{before}} \end{array}$$

where *C*_*before*_ and *C*_*after*_ mean the complexity indices before and after the flight task.

### 2.7. Statistics

The data were tested for normality first. As the data were not normally distributed, the nonparametric Mann–Whitney *U*-test was used to test whether there was significant difference between the two states, Sober (before the task) and MF (after the task) on each scale factor. For analyses that produced significant inter-group differences, a *post-hoc* test (Bonferroni *p*-value) was performed to determine the specific nature of the differences between the two states.

All statistical analyses above were carried out using R (R Core Team, 2021), and a significant level was set to 0.05.

## 3. Results

### 3.1. MSE analysis of alpha rhythm from spontaneous EEG (SPON-α)

**Figure 2:**
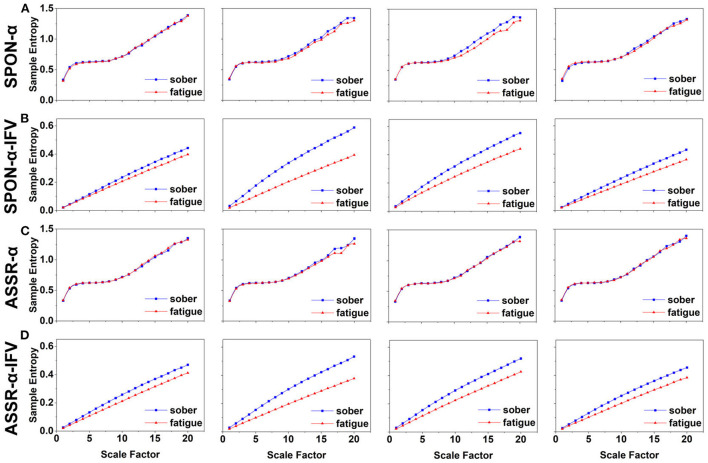
MSE analysis of SPON-α **(A)**, ASSR-α **(B)**, SPON-α-IFV **(C)**, and ASSR-α-IFV **(D)** on frontal lobe (channel F3) from four exemplary subjects. The blue and red lines represent the Sober and MF states, respectively. See Section 2.5 for the definitions of terms.

**Figure 3:**
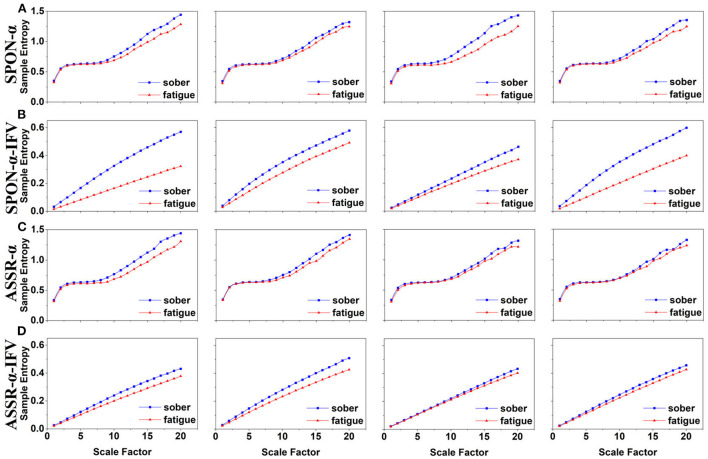
MSE analysis of SPON-α **(A)**, ASSR-α **(B)**, SPON-α-IFV **(C)**, and ASSR-α-IFV **(D)** on parietal lobe (channel P4) from four exemplary subjects. The blue and red lines represent the Sober and MF states, respectively. See Section 2.5 for the definitions of terms.

**Figure 4:**
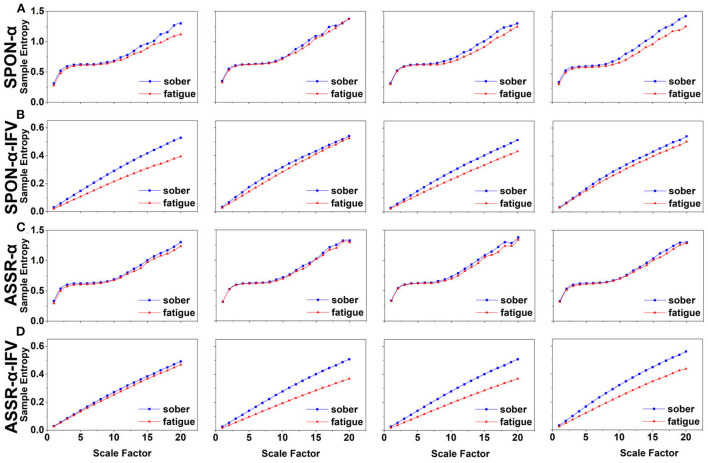
MSE analysis of SPON-α **(A)**, ASSR-α **(B)**, SPON-α-IFV **(C)**, and ASSR-α-IFV **(D)** on temporal lobe (channel T4) from four exemplary subjects. The blue and red lines represent the Sober and MF states, respectively. See Section 2.5 for the definitions of terms.

**Figure 5:**
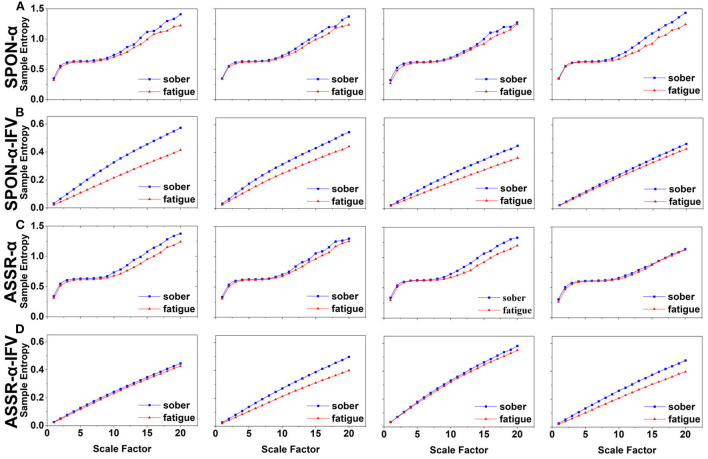
MSE analysis of SPON-α **(A)**, ASSR-α **(B)**, SPON-α-IFV **(C)**, and ASSR-α-IFV **(D)** on occipital lobe (channel O1) from four exemplary subjects. The blue and red lines represent the Sober and MF states, respectively. See Section 2.5 for the definitions of terms.

**Figure 6:**
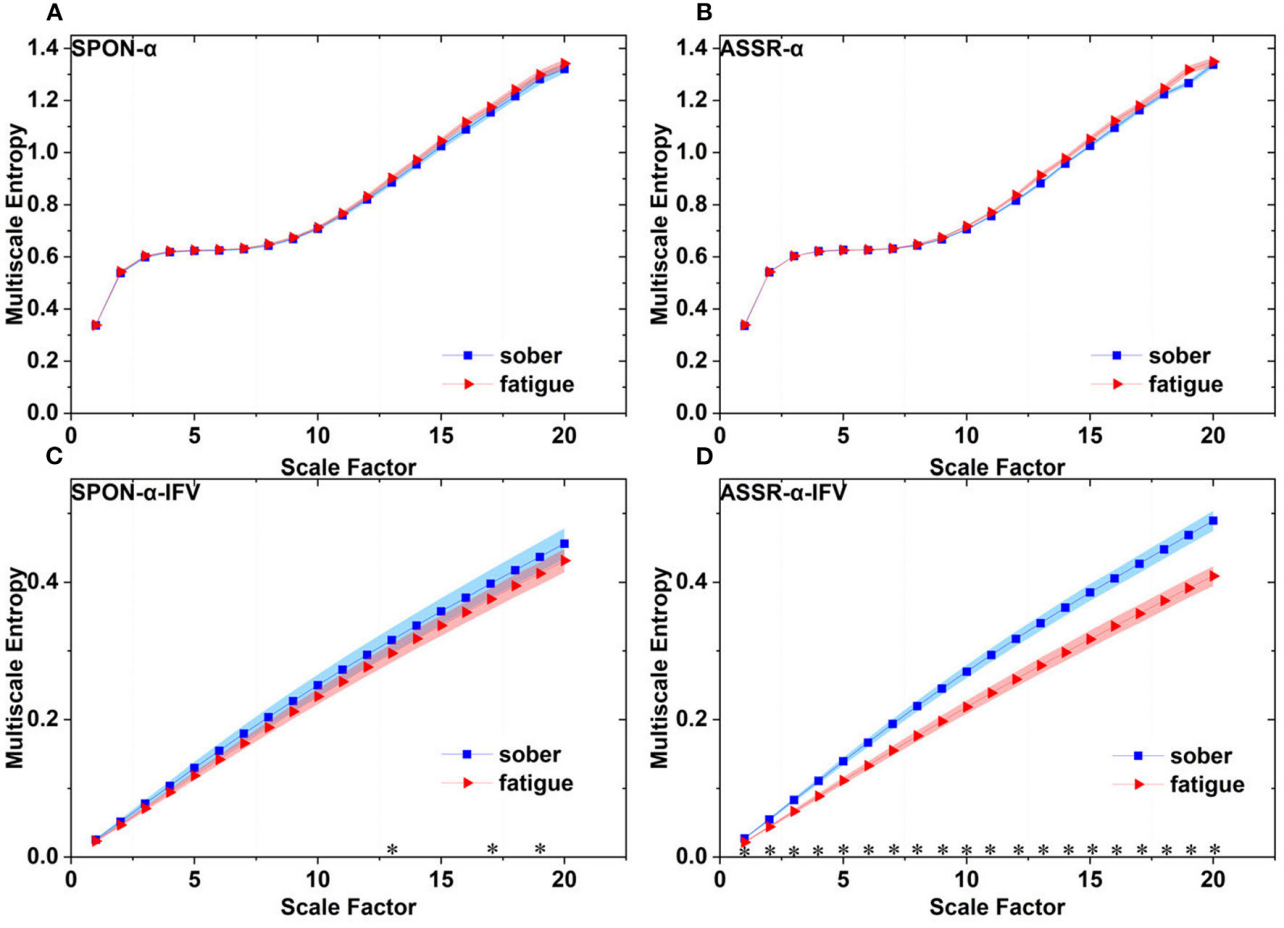
Comparison of MSE from frontal lobe (channel F3) on: **(A)** alpha rhythm from spontaneous EEG (SPON-α), **(B)** alpha rhythm from ASSR (ASSR-α), **(C)** IFV in alpha rhythm from spontaneous EEG (SPON-α-IFV), and **(D)** IFV in alpha rhythm from ASSR (ASSR-α-IFV). The sign * indicates significance at *p* < 0.05. Shades indicate the standard errors.

**Figure 7:**
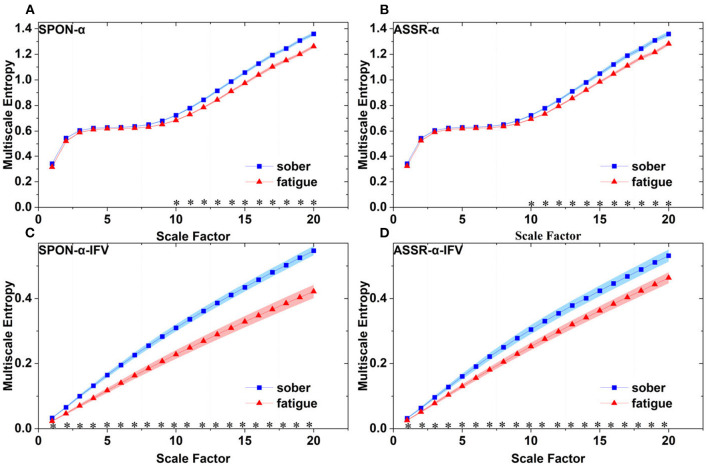
Comparison of MSE from parietal lobe (channel P4) on: **(A)** alpha rhythm from spontaneous EEG (SPON-α), **(B)** alpha rhythm from ASSR (ASSR-α), **(C)** IFV in alpha rhythm from spontaneous EEG (SPON-α-IFV), and **(D)** IFV in alpha rhythm from ASSR (ASSR-α-IFV). The sign * indicates significance at *p* < 0.05. Shades indicate the standard errors.

**Figure 8:**
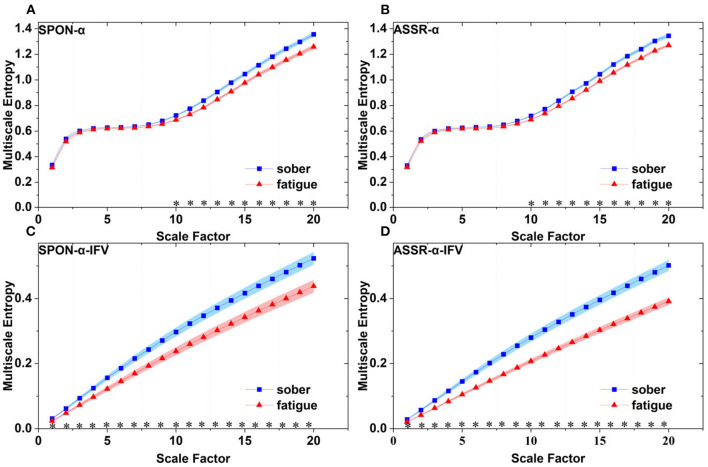
Comparison of MSE from frontal lobe (channel T3) on: **(A)** alpha rhythm from spontaneous EEG (SPON-α), **(B)** alpha rhythm from ASSR (ASSR-α), **(C)** IFV in alpha rhythm from spontaneous EEG (SPON-α-IFV), and **(D)** IFV in alpha rhythm from ASSR (ASSR-α-IFV). The sign * indicates significance at *p* < 0.05. Shades indicate the standard errors.

**Figure 9:**
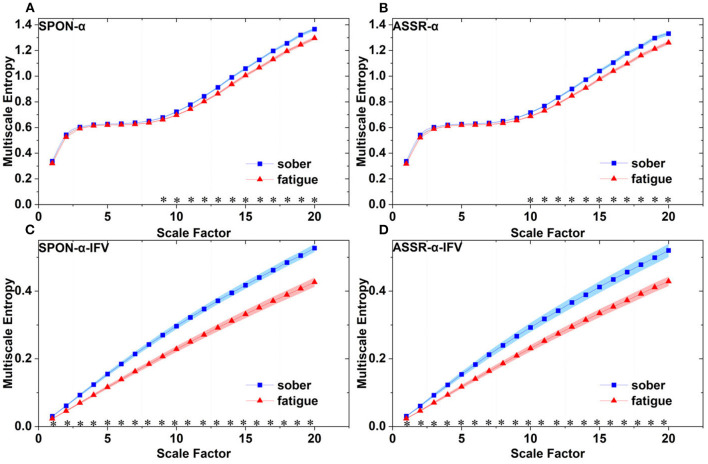
Comparison of MSE from occipital lobe (channel O1) on: **(A)** alpha rhythm from spontaneous EEG (SPON-α), **(B)** alpha rhythm from ASSR (ASSR-α), **(C)** IFV in alpha rhythm from spontaneous EEG (SPON-α-IFV), and **(D)** IFV in alpha rhythm from ASSR (ASSR-α-IFV). The sign * indicates significance at *p* < 0.05. Shades indicate the standard errors.

We analyzed four examples of MSE of SPON-α time series on frontal (Figure 2A), parietal (Figure 3A), temporal (Figure 4A), and occipital (Figure 5A) lobes. To quantify the differences among the four methods, we analyzed all subjects and carried out statistical analysis. Frontal lobe (Figure 6A), parietal lobe (Figure 7A), temporal lobe (Figure 8A), and occipital lobe (Figure 9A) show the group comparisons between Sober and MF states of MSE of SPON-α time series. In Figure 6A, the two MSE values of the two states on each scale are not significantly different (*p*>0.05, Mann–Whitney *U*-test). In Figures 7A, 8A, the two MSE values of the two states on scales from 10 to 20 are significantly different (*p* < 0.05, Mann–Whitney *U*-test). In Figure 9A, the two MSE values of the two states on scales from 9 to 20 are significantly different (*p* < 0.05, Mann–Whitney *U*-test).

### 3.2. MSE analysis of IFV in alpha rhythm from spontaneous EEG (SPON-α-IFV)

We analyzed four examples of MSE of SPON-α-IFV time series on frontal (Figure 2B), parietal (Figure 3B), temporal (Figure 4B), and occipital (Figure 5B) lobes. To quantify the differences among the four methods, we analyzed all subjects and carried out statistical analysis. Frontal lobe (Figure 6C), parietal lobe (Figure 7C), temporal lobe (Figure 8C), and occipital lobe (Figure 9C) show the group comparisons between Sober and MF states of MSE of SPON-α-IFV time series. In Figure 6C, the two MSE values of the two states on each scale are significantly different on scale 13, 17, 19 (*p* < 0.05, Mann–Whitney *U*-test). In Figures 7C, 8C, 9C, the two MSE values of the two states on all scales are significantly different (*p* < 0.05, Mann–Whitney *U*-test).

### 3.3. MSE analysis of alpha rhythm from ASSR (ASSR-α)

We analyzed four examples MSE of ASSR-α time series on frontal (Figure 2C), parietal (Figure 3C), temporal (Figure 4C), and occipital (Figure 5C). To quantify the differences among the four methods, we analyzed all subjects and carried out statistical analysis. Figures 6B, 7B, 8B, 9B show the group comparisons between Sober and MF states of MSE of ASSR-α time series. In Figure 6B, the two MSE values of the two states on each scale are not significantly different (*p*> 0.05, Mann–Whitney *U*-test). In Figures 7B, 8B, 9B, the two MSE values of the two states on scales from 10 to 20 are significantly different (*p* < 0.05, Mann–Whitney *U*-test).

### 3.4. MSE analysis of IFV in alpha rhythm from ASSR (ASSR-α-IFV)

We analyzed four examples of MSE of ASSR-α-IFV time series on frontal (Figure 2D), parietal (Figure 3D), temporal (Figure 4D), and occipital (Figure 5D) lobes. To quantify the differences among the four methods, we analyzed all subjects and carried out statistical analysis. Frontal lobe (Figure 6D), parietal lobe (Figure 7D), temporal lobe (Figure 8D), and occipital lobe (Figure 9D) show the group comparisons between Sober and MF states of MSE of ASSR-α-IFV time series. In Figures 6D, 7D, 8D, 9D, the two MSE values of the two states on each scale are significantly different on all scales (*p* < 0.001, Mann–Whitney *U*-test).

### 3.5. Comparison of four methods

**Figure 10:**
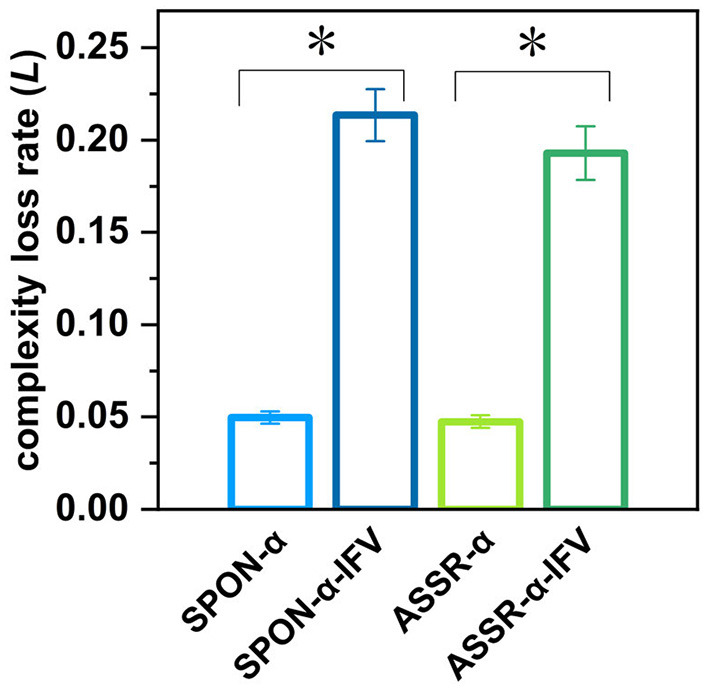
Complexity loss rates of SPON-α, ASSR-α, SPON-α-IFV, and ASSR-α-IFV from all subjects and channels. See Section 2.5 for the definitions of terms. The sign * indicates significance at *p* < 0.05.

Figure 10 shows that there was significant difference between the complexity loss rates of SPON-α and SPON-α-IFV (*p* = 7.41 × 10^−18^, Mann–Whitney *U*-test), and between ASSR-α and ASSR-α-IFV (*p* = 1.89 × 10^−17^, Mann–Whitney *U*-test). No significant differences were found between the complexity loss rates of SPON-α and ASSR-α (*p* = 0.62, Mann–Whitney *U*-test) was found either and between SPON-α-IFV and ASSR-α-IFV (*p* = 0.20, Mann–Whitney *U*-test).

We compared the time consumption of calculating MSE of SPON-α, SPON-α-IFV, ASSR-α, ASSR-α-IVF on a desktop computer (Laptop computer, Windows 10, Intel Core i7-9750H CPU, 2.60 GHz). The average MSE calculation time of ASSR-α-IVF was 79.033 s, which is ~8.9 times as long as that of the conventional MSE on SPON-α method (Table 1).

**Table 1:** Time consumption comparison of MSE calculation.

**Method**	**SPON-α**	**SPON-α-IFV**	**ASSR-α**	**ASSR-α-IFV**
Time consumption ± standard deviation(s)	8.894 ± 0.424	76.304 ± 5.798	8.928 ± 0.574	79.033 ± 3.985

## 4. Discussion

In this study, we have focused on the hypothesis that MF can induce the complexity loss. EEG signals were recorded from resting state with and without ASSR stimulus. From the EEG signals, alpha rhythms were extracted using a filter and then IFV in alpha rhythms was calculated using Hilbert transform, and analyzed using the MSE method. Finally, the complexity loss rate (defined in Equation 5) of four types of time series, SPON-α, SPON-α-IFV, ASSR-α, and ASSR-α-IFV, between Sober and MF states were calculated and compared.

MF impairs a variety of cognitive functions (Mast and Heimstra, 1964) and according to the complexity loss theory (Lipsitz and Goldberger, 1992), should induce lower complexity in EEG. However, the few studies that cover this topic report inconsistent results (Chen et al., 2018; Zhang et al., 2021). In this study, we have applied two strategies in the attempt to test the hypotheses that MF can also cause the complexity loss. Our results of MSE analysis on SPON-α are in accordance with the work (Chen et al., 2018) in that on some scales, there are complexity loss after cognition demanding tasks (watching 3DTV in Chen et al.'s work or flying a simulated plane in this study). In addition, we have shown that IVF in alpha rhythms either from spontaneous EEG or from ASSR, the complexity loss is more obvious showing higher detectability. While in Zhang et al. (2021)'s study, MF induced by days of intellectual competition caused increased complexity and may have involved unexpected factors.

Alpha frequency has bee associated with age (Grandy et al., 2013), visual perception (Battaglini et al., 2020), memory performance (Klimesch et al., 1993), cognitive preparedness (Angelakis et al., 2004), speed of reaction (Klimesch et al., 1996). Alpha peak frequency varies constantly, and the variation does not represent random fluctuations but instead constitutes the basis of an adaptive mechanism mirroring the activation level of neural populations (Mierau et al., 2017). Previously, we have shown that the EEG alpha rhythm changes its peak frequency constantly and the MSE analysis of the instantaneous frequency variation (IVF) can be used to show the complexity loss of the brain in sleep comparing to in wakefulness (Li et al., 2021).

In Li et al. (2021)'s work, the reason for using MSE analysis on IFV in spontaneous alpha rhythm or delta wave instead of on the brain wave itself is that the MSE algorithm suffers from the artifact of the carrying frequency of the brain waves. In this study, we have also shown the IFV in alpha rhythm either from spontaneous EEG or from ASSR has much higher separation power. The possible reason is that the alpha amplitude is more subject to noise, while the IFV is frequency modulated, which is more stable. It is similar to the higher quality of frequency-modulated (FM) radio signal comparing to the amplitude-modulated (AM) signal.

The ASSR is a type of event related potential which is often used to test the integrity of auditory pathways and the capacity of these pathways to generate synchronous activity at specific frequencies (Brenner et al., 2009). In our study, MSE analysis of ASSR-α-IFV significantly increase the complexity loss effect than that of ASSR-α. We have chosen 80 Hz instead of 90 Hz to avoid mains interference around 100 Hz, which is the harmonic of 50 Hz.

### 4.1. Limitations

In the current study, we focused on the complexity loss of IFV in alpha rhythm from spontaneous EEG and auditory stimulated EEG, and in the future, we need to investigate the other frequency bands.

## 5. Conclusion

In this study, we have shown mental fatigue can cause complexity loss of EEG, by analyzing the multiscale entropy of the IFV in alpha rhythm extracted from spontaneous EEG and ASSR. This complexity loss phenomenon can be largely concealed with conventional MSE analysis on spontaneous EEG. This implicates that the MSE analysis on IFV in brain rhythms can be a promising method to reveal the complexity of a physiological process.

## Data availability statement

The original contributions presented in the study are included in the article/supplementary material, further inquiries can be directed to the corresponding author/s.

## Ethics statement

The studies involving human participants were reviewed and approved by the Institutional Ethical Review Board at the Air Force Medical University. The patients/participants provided their written informed consent to participate in this study.

## Author contributions

YZ: study ideation and planning, statistical analyses, and writing. YL and SZ: study ideation, data collection, and writing. CZ: data processing. EL: writing. CT: ideation, planning, and writing. KX: study ideation, planning, and writing supervision of all phases of the study. All authors contributed to the article and approved the submitted version.

## Funding

This work was supported by Air Force Medical University (2020rcfcxkn).

## Conflict of interest

The authors declare that the research was conducted in the absence of any commercial or financial relationships that could be construed as a potential conflict of interest.

## Publisher's note

All claims expressed in this article are solely those of the authors and do not necessarily represent those of their affiliated organizations, or those of the publisher, the editors and the reviewers. Any product that may be evaluated in this article, or claim that may be made by its manufacturer, is not guaranteed or endorsed by the publisher.
